# The Prevention of Suicide in Older Military Veterans

**DOI:** 10.3390/bs15030379

**Published:** 2025-03-17

**Authors:** Joshua Levine, Leo Sher

**Affiliations:** 1 Veterans’ Administration New York Harbor Healthcare System, Brooklyn, NY 11209, USA; jpl2158@columbia.edu; 2 School of Social Work, Columbia University, New York, NY 10027, USA; 3 James J. Peters Veterans’ Administration Medical Center, Bronx, NY 10468, USA; 4 Department of Psychiatry, Icahn School of Medicine at Mount Sinai, New York, NY 10029, USA

**Keywords:** suicide, public health, depression, stress, military veterans, older

## Abstract

Suicidal behavior among older military veterans is an important medical and social problem. The goal of this literature review is to discuss this underappreciated issue and identify suicide preventive interventions that can be utilized with the older military veteran population. Older veterans experience psychiatric, medical, and social problems associated with their age and/or military experience that can contribute to suicide risk. These problems include relationship losses through death or estrangement, depression, cognitive decline, loneliness, isolation, frailty, mobility issues, and chronic pain. Therefore, older veterans face a unique set of challenges. Suicide prevention in older veterans should take a multipronged approach which includes screening for suicidality, management of psychiatric and medical disorders, social assistance, safety planning, lethal means restriction, and involving family members in the veteran’s healthcare. Family members should be included in the safety planning process when possible. Gatekeeper training programs can be utilized to train individuals who are working with older veterans to reduce suicides amongst this age group.

## 1. Introduction: Geriatric Suicide—A Major Public Health Problem

The World Health Organization (WHO) reported that nearly one in six people will be 60 years or older by the year 2030 (World Health Organization, 2023). Suicide among the older adult population is a multinational problem. In 2019, 256,953 (36.53%) of the 703,220 people who were estimated to die by suicide globally were aged 60 and older (World Health Organization, 2020). It is possible that suicide rates are underestimated. Many suicide deaths may be incorrectly documented as “unnatural” or “undetermined” deaths (Sher & Oquendo, 2023; Tøllefson et al., 2015). Actual suicide rates may be 10–50% higher than reported. This makes suicide prevention efforts among the older population a crucial undertaking. Older adults experience problems associated with their age that can contribute to suicide risk. These problems consist of relationship losses through death or estrangement, mental health disorders (i.e., anxiety or depression), medical problems, cognitive decline, loneliness, isolation, frailty, mobility issues, and chronic pain (Conwell et al., 2011; O’Malley et al., 2020). Older persons are also at risk for neglect and elder abuse (i.e., physical, psychological, sexual, or financial) (Cooper et al., 2008; Yunus et al., 2019). For example, one review found that 6% of older people reported abuse in the last month, and nearly 25% of vulnerable older persons were at risk for abuse (Cooper et al., 2008).

Oftentimes, a considerable amount of attention is given in cases of adolescent suicide (Carballo et al., 2020; S. Kim et al., 2024; Zartaloudi, 2024). This may be because of the thought of years of potential life lost. From a humanitarian standpoint, geriatric suicide is an important problem, and should be a focus of suicide prevention efforts.

Suicide among older people, including older veterans, does not receive enough attention. The goal of this paper is to discuss this underappreciated issue and identify suicide preventive interventions that can be utilized with the older military veteran population.

## 2. Definitions

As noted above, this review is dedicated to the issue of suicidal behavior among older veterans. Suicidal behavior suggests suicide death but also includes different kinds of suicide attempts that range from highly lethal attempts to low-lethality attempts that may occur during a psychosocial crisis (Naguy et al., 2020; Sher & Oquendo, 2023; Turecki et al., 2019). A suicide attempt is commonly defined as a self-destructive act carried out with some degree of intent to end one’s life. Suicidal ideation is a term used to describe suicidal thoughts (Harmer et al., 2024). Some suicide ideation definitions incorporate suicide planning thoughts, while others regard planning to be a separate phase. Suicidal ideation is associated with both suicide attempts and deaths and is a significant risk factor for future suicide attempts (Baca-Garcia et al., 2011; Sher & Oquendo, 2023). Suicidal ideation and suicidal behaviors can be conceptualized as taking place over a continuum from suicidal ideation, development of a suicide plan, nonfatal suicide attempt(s), or death from suicide, although the evolution from one to the other is not necessarily linear (Baca-Garcia et al., 2011). Multiple risk factors such as recent onset of suicidal ideation, the presence and recent onset of suicide plan, extreme risk taking, and failure to answer questions about the characteristics of one’s suicidal thoughts predict transition from suicidal ideation to suicide attempt (Nock et al., 2018).

In the United States (U.S.), a military veteran is an individual who has served in the Armed Forces, including the Reserve (United States Census Bureau, 2021). Combat veterans are individuals who served in a conflict/war zone while in the military. In many countries, individuals are classified as military veterans if they served during wartime and/or participated in combat operations; i.e., only combat veterans are regarded as veterans (EUROMIL, 2022).

The U.S. National Institute on Aging defines older adults as people aged 65 or older (National Institutes of Health, 2024). However, definitions of older adulthood differ. For example, an older person is defined by the United Nations as an individual who is over 60 years of age (The UN Refugee Agency, 2024). It has also been suggested that “While age 60 was considered “old” in many peoples’ grandparents’ time, 80 is the median age considered “old” today” (Stagwell Global, 2024). For the purposes of this article, we assume that older adulthood is the stage of life usually beginning around age 65 and is characterized by different physical, cognitive, and social changes associated with aging.

## 3. Suicide Among the Military Veteran Population

As per the recent reports released by the U.S. Census Bureau, there are around 18 million military veterans and 2.1 million active-duty and Reserve service members in the U.S. (United States Census Bureau, 2020). More than 6% of the US population has served or is currently serving in the Armed Forces.

Suicide amongst the military veteran population is a significant public health problem (Department of Veterans Affairs, 2023; Levine & Sher, 2021c; Shen et al., 2016). Before 2000, the suicide rates among military servicemembers and veterans were lower than those among civilians. However, these rates have increased over the last 20 years and exceed the current rate among civilians (Moore et al., 2023). The National Veteran Suicide Prevention Annual Report revealed that 6278 U.S. military veterans died by suicide in 2020, and this number raised to 6392 in 2021 (Department of Veterans Affairs, 2023). This accounts for an increase of 17.2 to 17.5 average veteran suicides per day from 2020 to 2021 (Department of Veterans Affairs, 2023). Unfortunately, the COVID-19 pandemic may account for an increase in veteran suicide rates (Levine & Sher, 2021b).

Military veterans are at greater risk for medical problems, psychiatric disorders, and psychosocial stressors as compared to the general population (Bruce, 2010; Levine & Sher, 2021c; Pew Research Center, 2019; Rice & Sher, 2012). Research indicates that the presence of these conditions can increase suicidality in military veterans (Bruce, 2010).

## 4. What Makes Older Veterans Suicidal?

Older veterans may face a unique set of challenges as compared to their younger counterparts. They have more severe medical disorders, psychiatric problems, and psychosocial difficulties than younger veterans. The presentation of psychiatric and physical disorders may be different in the elderly population in comparison to the younger individuals. Considerable evidence suggests that older veterans are at elevated risk for suicide (Kaplan et al., 2012; Mills et al., 2013; Sullivan et al., 2023; Zivin et al., 2007).

### 4.1. Psychiatric Disorders

Many military veterans have unique experiences that the general population will not encounter. These circumstances might consist of combat exposure, being exposed to a chemical or toxin (i.e., Agent Orange or radiation), or deployment to a harsh terrain (i.e., desert or jungle). Combat exposure could lead to posttraumatic stress disorder (PTSD), physical injury, or being captured as a prisoner of war. Research shows that approximately 14–16% of U.S. military personnel who were deployed have a PTSD diagnosis (Gates et al., 2012). Comparatively, PTSD only affects about 7–8% of the U.S. general population (Gates et al., 2012). Among veterans older than 60, the prevalence of PTSD in combat veterans is 16.9%, and it is 5.5% among non-combat veterans (Goldberg et al., 2016). Combat exposure is not the only factor that contributes to PTSD in military veterans. Unfortunately, military veterans can also be victims of Military Sexual Trauma (MST). MST refers to sexual harassment or sexual assault that was experienced during military service (Kimerling et al., 2007). Additionally, military veterans who have never had a combat deployment might experience an incident on base that can result in PTSD, such as accidental death of a counterpart. PTSD has been shown to increase suicide risk in veteran populations (Bruce, 2010; Pompili et al., 2013; Sher, 2024b). Most studies, but not all studies, indicate that combat veterans are at higher risk for suicide than non-combat veterans or the general population (Bullman & Schneiderman, 2021; Schoenbaum et al., 2014; Shen et al., 2016; Sher, 2024b). For example, one study found that Operation Enduring Freedom and Operation Iraqi Freedom (OEF/OIF) war veterans had elevated suicide rates for the first 4 years after their deployment, in comparison with veterans who had not been deployed (Shen et al., 2016). It is a debatable issue that veterans who have had combat deployments are at the highest risk of suicide. One study reviewed data of 3.9 million U.S. military personnel who served in OEF/OIF (Reger et al., 2015). The study found that military personnel who were deployed were not at higher risk for suicide, as compared to those who were not deployed (Reger et al., 2015).

A meta-analysis and systematic review that reviewed eleven studies of veterans aged >65 years found that older veterans had a higher prevalence of alcohol and substance use disorders as compared to community estimates (Williamson et al., 2018). Veterans who experienced traumatic events earlier in their life might experience re-emerging PTSD symptoms during their geriatric years (Davison et al., 2016).

PTSD is not the only psychiatric disorder that is prevalent amongst military veterans. Seal et al. (2007) reviewed healthcare data of 103,788 U.S. military veterans who served in OEF/OIF (Seal et al., 2007). The researchers concluded that 25% (25,658) of these veterans had at least one mental health diagnosis consisting of either PTSD (13%), adjustment disorder (6%), anxiety disorder (6%), depression (5%), substance use disorder (5%), or other mental health disorder (12%) (Seal et al., 2007). Another study reviewed data of 821 United Kingdom (UK) OEF/OIF military veterans and identified the presence of the following mental health disorders: PTSD (4.8%), neurotic disorders (13.5%), depression disorders (11%), anxiety disorders (4.5%), somatization disorders (1.8%), and alcohol abuse (18%) (Iversen et al., 2009). All these psychiatric conditions are associated with suicidal ideation and behavior (Sher & Oquendo, 2023) and may increase suicide risk in older military veterans.

### 4.2. Medical Illnesses

Aging is associated with the development of multiple medical disorders including cardiovascular disease, metabolic conditions, chronic obstructive pulmonary disease, hearing loss, eye diseases, and pain syndromes including back and neck pain and osteoarthritis (Callahan et al., 2002; Fillit et al., 2016; Straus, 2001). All these conditions are associated with elevated suicide risk (Choi et al., 2023; Sher, 2024a). Medical issues that lead to functional limitations are associated with significantly increased suicide risk among the general population and are more prevalent among veterans ages 65 and older than in the general population (Bongar et al., 2017; Kaplan et al., 2007; Kaplan et al., 2012). All medical and neurological conditions, especially pain syndromes and traumatic brain injury (TBI), increase suicide risk in older veterans (Elman et al., 2013; Kirtley et al., 2020; Kuffel et al., 2023; Pakniyat-Jahromi et al., 2022; Rice & Sher, 2012). Higher lethality of suicide attempts among older adults is related to declines in physical condition (D. W. Kim et al., 2021).

Additionally, older veterans may have physical limitations, such as frailty, that make it difficult for them to ambulate to their healthcare appointments. One study found that frailty is associated with increased risk of suicide attempts and higher rates of suicide deaths in U.S. military veterans (Kuffel et al., 2023).

Suicidality in older veterans may be affected by combat-related injuries, medical disorders or a combination of both (Bullman & Kang, 1996; Kaplan et al., 2012; Rice & Sher, 2012). A 19-year post-War World II study of American veterans with spinal cord injury showed that suicide was the third most common cause of death, ranking behind only renal failure and secondary amyloidosis (Nyquist & Bors, 1967). In American Vietnam veterans, sustaining wounds that required hospitalization or sustaining two or more wounds was observed to increase the risk of suicide by 1.2- and 1.6-fold, respectively (Bullman & Kang, 1996). Combat or non-combat TBIs may increase suicide risk among military veterans (Brenner et al., 2011; Campbell-Sills et al., 2020; Silver et al., 2001).

### 4.3. Psychosocial Stressors

Older veterans have a lot of strengths and significant life experience to cope with challenges that are frequent in later life. However, psychosocial difficulties may negatively affect the mental and/or physical health of some older veterans and result in suicidal behavior (Beydoun et al., 2024; Fletcher et al., 2023; Wilson et al., 2018; Wirtz & von Känel, 2017). The VA Behavioral Health Autopsy Program (BHAP) reviewed 2545 U.S. military veteran suicides between 2019 and 2021 and found that 24.7% of veterans who died by suicide experienced financial loss, 12.6% had legal problems or arrest, and 11.5% were at risk for homelessness (Department of Veterans Affairs, 2023).

In older veterans, psychosocial stressors can be related to multiple life problems, including loneliness, loss of loved ones, financial difficulties, poor living conditions/homelessness, traumatic life events, low neighbor cohesion, and lower subjective and objective social status (Wilson et al., 2018; Wirtz & von Känel, 2017). A review of loneliness and social isolation of military veterans has demonstrated that older veterans present unique experiences of loneliness and social isolation (Fletcher et al., 2023; Wilson et al., 2018). The authors have also noted that there are differences between military cultures of the Armed Forces of different nations which may affect veterans’ perception of social isolation and loneliness.

Population-based studies indicate that the risk for suicide grows with an increase in the number of risk factors present. In other words, when more risk factors are present at any one time, the more likely it is that they indicate an increased risk for suicidal behaviors at that time.

## 5. Prevention of Suicidal Behavior in Older Veterans

Suicide prevention efforts should take a multipronged approach when addressing the needs of older veterans. This approach should incorporate community education through gatekeeper training programs, involving family members in the veteran’s healthcare, the use of technology, lethal means restriction, safety planning, treatment and management of medical and psychiatric disorders, the use of screening instruments and interviews, and the interdisciplinary team framework.

## 6. Diagnosis/Recognition

A psychiatric interview performed by a competent medical professional is the foundation of suicide risk evaluations (Lake et al., 1984; Sher, 2011). Healthcare professionals need to be able to estimate suicide risk based on all available information including patient answers to direct and indirect questions, known risk factors, information on how the patient behaved under similar circumstances in the past, and collateral information. It is vital to educate medical professionals that not all suicidal patients including older veterans report suicidal ideation, intent, or plan to clinicians. Screening questionnaires may help identify veterans at risk for suicidal behavior (National Institutes of Health, 2015). It has been suggested that a brief screening tool can identify individuals at risk for suicide, more reliably than leaving the identification up to a clinician’s judgment (The Joint Commission, 2016). The Patient Health Questionnaire (PHQ-9) is a screening instrument that can assess suicide risk. One study that used the PHQ-9 found that individuals who expressed thoughts of self-harm or death were 10× more likely to attempt suicide than those who did not report those thoughts (Kroenke et al., 2001). Another screening tool that can be utilized to assess suicide risk is the Columbia Suicide Severity Rating Scale (C-SSRS). Researchers evaluated the C-SSRS over three multi-site studies and found it to be effective in assessing for suicidal ideation and behavior in clinical and research settings (Posner et al., 2011).

Once a veteran screens positive on a brief screening tool, a comprehensive suicide risk assessment should be completed. The suicide risk assessment should incorporate suicidal ideation, suicide plan, means available, preparatory acts, level of intent, reasons for living/lying/dying, previous suicide attempts, warning signs, risk/protective factors, and collateral information (Fowler, 2012). When assessing suicidal ideation, it is important to obtain as much detail as possible about the frequency, duration and intensity of the ideation.

## 7. Interventions

### 7.1. The Treatment of Psychiatric Conditions and Management of Medical Disorders

Effective management of psychiatric disorders is the foundation of suicide prevention in any patient population including older veterans (Mann et al., 2005; Rihmer & Gonda, 2013; Sher & Oquendo, 2023). Different pharmacological and psychological treatment modalities can be used. Pharmacological interventions need to take into account the age and medical conditions of the veterans; i.e., doses of psychotropic medications need to be adjusted (Bednarczyk et al., 2022; Varma et al., 2010). Older adults may have greater medication effects compared to younger people given the same doses of drugs. Aging is usually associated with an increase in the proportion of body fat in the body, causing an increase in the elimination half-life of many psychotropic and non-psychotropic medications. It should be noted that older adults are at significantly higher risk of having pharmacodynamic drug–drug interactions due to polypharmacy than younger people (Varma et al., 2010). Pharmacodynamic interactions occur when two or more drugs influence each other’s effects at the cellular or molecular level (Varma et al., 2010).

Psychological treatment modalities should be carefully chosen specifically for the disorder that is being treated. For example, one pilot study found that prolonged exposure therapy was effective in treating PTSD in older veterans (Thorp et al., 2012). However, some researchers caution against the use of intensive exposure therapies with older adults (Owens et al., 2005). These interventions may trigger an autonomic arousal, which, combined with other medical comorbidities such as cardiovascular disease, could pose life-threatening consequences (Owens et al., 2005).

Treatment of medical disorders, especially conditions associated with pain, can also decrease suicide risk (Huang et al., 2022; Rice & Sher, 2012). Older veterans with chronic pain may have decreased mobility, which limits their ability to work or participate in daily activities, and successful treatment of these veterans may improve their mobility and consequently reduce suicidality.

### 7.2. Safety Planning

The suicide prevention safety plan is an effective tool that could be utilized in a crisis situation involving an older veteran (Matthieu et al., 2023; Stanley & Brown, 2012). It is a prioritized list of coping strategies that is tailored to an individual’s specific mental health needs. Mental health professionals should complete the suicide prevention safety plan synchronously and in collaboration with the veteran. The safety plan should not be completed if the individual is actively experiencing a crisis situation, and it should be completed more so as a preventative measure. Each step of the safety plan should be reviewed in detail, and the veteran should be encouraged to utilize their safety plan in a crisis situation. The safety plan should be periodically reviewed through the course of treatment and updated with any relevant changes.

A review of the literature found that safety plans were effective in preventing suicidal behavior (Nuij et al., 2021). The researchers mentioned that there was no evidence that suicide prevention safety plans reduced suicidal ideation (Nuij et al., 2021). They recommended the use of other interventions, such as cognitive behavioral therapy or dialectical behavior therapy, to treat suicidal ideation (Nuij et al., 2021).

### 7.3. Social Help

Older veterans who are experiencing psychosocial stressors should be provided with social help. As noted above, research shows that psychosocial stressors may increase suicidality (Pompili et al., 2013). Levine and Sher propose increasing the role of social workers in global suicide prevention efforts (Levine & Sher, 2020). Social workers can assist older veterans with obtaining government benefits, case management, care coordination, referrals to community resources (i.e., home healthcare), housing placement (i.e., assisted living or nursing home), and legal assistance (Levine & Sher, 2021a). In cases of elder abuse, financial exploitation, or neglect, social workers can help older veterans navigate the appropriate legal channels or get connected to government resources.

### 7.4. Family Education

Family members should be included in an older veteran’s healthcare, when applicable (Jazieh et al., 2018; Sandi et al., 2020; Shimange et al., 2024). Healthcare professionals can educate family members on lethal means restriction and include them in the safety planning process. Family members can assist with or provide confirmation of the disposal of lethal means, such as the disposal of expired or unused medications. They can also safely administer medications to older veterans with the use of daily pill organizers or medication lockboxes. In cases where older veterans are no longer able to live independently, social workers can work with family members to assist with placement in an assisted living or nursing home setting.

### 7.5. Public Education and Role of Gatekeepers

Public education can be effective in suicide prevention efforts with older military veterans (Levine & Sher, 2020; Mann et al., 2005). Healthcare professionals can educate the public on suicide risk factors and warning signs with the use of gatekeeper training programs or lectures (Levine & Sher, 2020). If community members have a general understanding of the scope of suicide amongst veterans, they can better screen the veteran and connect them to the appropriate resources. Employing the use of social workers to educate the public has been proposed as a means to decrease the overall suicides in a given area (Levine & Sher, 2020).

Gatekeeper training is an effective suicide prevention intervention (Mann et al., 2005). Gatekeepers are people who are trained to identify suicide risk factors and connect suicidal persons to appropriate services (Mann et al., 2005). Question, Persuade, and Referral (QPR) is one example of a gatekeeper training program (Quinnett, 1995). Researchers evaluated the QPR program amongst clinical and non-clinical U.S. Department of Veterans Affairs staff (Matthieu et al., 2008). The study found a significant increase in self-efficacy and knowledge amongst both clinical and non-clinical staff, with non-clinical staff having larger effect sizes in both areas (Matthieu et al., 2008). Gatekeeper training programs can be utilized to train individuals who are working with older veterans as a way to reduce suicides amongst this age group.

### 7.6. Lethal Means Restriction

Research shows that lethal means restriction is one of the most effective societal interventions for preventing suicides (Yip et al., 2012). In September 1998, the United Kingdom introduced new legislation to limit the size of over-the-counter analgesic packs (salicylates, paracetamol, and their compounds) in an effort to reduce suicides (Hawton et al., 2004). This resulted in a 22% decrease in suicide deaths from paracetamol and salicylate overdoses in the succeeding year, with the trend continuing for three years after the legislation (Hawton et al., 2004). In 2006, Israel created the Israeli Defense Force (IDF) Suicide Prevention Program, which was designed to reduce firearm-related suicides amongst military personnel (Shelef et al., 2015). One of the aspects of this program was that IDF soldiers were required to lock their firearms in storage when on leave (Shelef et al., 2015). Suicide rates among IDF soldiers dropped by nearly 50% from 2006 to 2014 (Shelef et al., 2015).

Military veterans often have access to firearms and knowledge on how to use them from their military experience (Levine & Sher, 2021c). Firearm ownership in the U.S. is more prevalent amongst the veteran population (45% ownership) compared to the non-veteran population (19% ownership) (Department of Veterans Affairs, 2023). In 2021, the U.S. Department of Veterans Affairs reported that 73.4% of suicides among male veterans and 51.7% of suicides among female veterans involved the use of a firearm (Department of Veterans Affairs, 2023). Lethal means restriction can be utilized in cases of firearm ownership. Veterans can be encouraged to relinquish their firearms through a law enforcement agency. If the veteran is unwilling to give up their firearm, they can be encouraged to utilize a gunlock and/or gun locker. Ammunition can be stored separately from the firearm, ideally in an inconvenient location, or disposed of altogether. Firearms can be disassembled, so they cannot be easily used in a suicide attempt.

Older people often have access to a lot of medications due to medical issues related to old age. Lethal means restriction can also be utilized with medications. Medications can be portioned in weekly pill organizers, and excess medications can be stored in a medication lockbox. A family member or friend can administer the medications to the veteran as prescribed. Additionally, prescribers can limit the amount or refills or quantity of pills. Veterans can also be given information on where to dispose of unused or expired medications, such as at the local pharmacy or with the use of Deterra pouches. In cases when veterans are prescribed opioid medications, they should be provided with Narcan kits and information on how to use these kits.

### 7.7. Technology

Technology can play an important role in suicide preventive efforts with older military veterans. Following the COVID-19 outbreak, telehealth interventions have been increasingly used to increase access to healthcare (Bracken et al., 2024; Haderlein et al., 2024; Woon et al., 2024). Although it is preferred for healthcare services to be delivered in-person, especially for individuals who are at high risk for suicide, the use of technology can provide support to older veterans with mobility issues or who reside in rural areas. One study reviewed the efficacy of telehealth interventions in suicide prevention and found that telehealth was effective in reducing suicidal ideation, suicide reattempts, and suicide rates (Shoib et al., 2024). The researchers recommended that mental health professionals provide monthly telehealth outreaches for at least 12 months following an index suicide attempt (Shoib et al., 2024). A recent study in the Bronx, New York, showed that telehealth-delivered horticultural therapy significantly reduces risk factors for suicidality among veterans including stress, depression, and loneliness (Meore et al., 2024). Horticultural therapy utilizes gardening as a way to promote physical, emotional, mental and social well-being, which is especially important in older veterans (Meore et al., 2024).

It has been recommended to use social media in suicide preventive efforts (Levine & Sher, 2020; Robinson et al., 2016). Social media can provide support through online forums or support groups, identify older veterans who make suicidal statements over the internet, or reach individuals who are otherwise difficult to engage (Robinson et al., 2016). On the contrary, some researchers suggest that social media could increase suicide risk by posing a risk for contagion, increasing isolation, or normalizing suicidal behaviors (Robinson et al., 2016). Mental health providers should provide education on the positive and negative impacts of social media when working with older veterans (Levine & Sher, 2020).

Mobile apps that are designed for mental health can be utilized to reduce suicide risk, as they are publicly available and highly scalable (Melia et al., 2020). The Department of Veterans Affairs has created several mobile apps to support the overall well-being of U.S. military veterans. These apps consist of self-care activities, mindfulness or meditation exercises, physical exercises (i.e., Tai Chi, yoga), smoking cessation, suicide prevention safety plans, and resources related to various medical or mental health disorders (Department of Veterans Affairs, 2023).

Technology can also be incorporated through wearable alert systems and smart home technology that can actively and passively monitor physiological, emotional, behavioral and cognitive changes that may lead to suicidal behavior (Stanley & Mann, 2020). Older veterans need to be educated regarding the use of modern technology.

### 7.8. Interdisciplinary Approach

An interdisciplinary approach should be utilized when working with older military veterans at risk for suicide (Levine & Sher, 2021a). This approach should incorporate the collaboration of mental health professionals, medical professionals, caseworkers, lawyers, and other community professionals involved in the veteran’s care (Holmes et al., 2020). One study evaluated an interdisciplinary care management intervention for individuals aged 60 years or older with minor or major depression (Alexopoulos et al., 2009). Study participants received case management and treatment services from social workers, nurses, psychologists and physicians over a two-year span (Alexopoulos et al., 2009). The researchers found that participants who received the interdisciplinary care management intervention had a 2.2-fold decrease in suicidal ideation, as compared to those who did not receive the intervention (Alexopoulos et al., 2009).

## 8. Conclusions

Suicide among the geriatric population is an underappreciated topic, particularly among military veterans. Older veterans may be hesitant to address their mental health issues due to generational influences, such as stigma associated with having a mental illness. Challenges that older male and female veterans may experience may include low mobility, frailty, isolation, loneliness, loss of loved ones, and cognitive decline. Researchers suggest that there is a paucity of data on suicide prevention research for military veterans aged 65 and older (Sullivan et al., 2023). Older veterans with suicidal behavior should not be neglected. It has been argued that it is a moral obligation for society to ensure that older persons are provided with comfort, health and security (Upadhyaya & Sher, 2019).

## Data Availability

Not applicable.
